# Evolution through segmental duplications and losses: a Super-Reconciliation approach

**DOI:** 10.1186/s13015-020-00171-4

**Published:** 2020-05-26

**Authors:** Mattéo Delabre, Nadia El-Mabrouk, Katharina T. Huber, Manuel Lafond, Vincent Moulton, Emmanuel Noutahi, Miguel Sautie Castellanos

**Affiliations:** 1 Département d’informatique (DIRO), Université de Montréal, Québec, Canada; 2 School of Computing Sciences, University of East Anglia, Norwich, UK; 3 Department of Computer Science, Université de Sherbrooke, Sherbrooke, Canada

**Keywords:** Gene tree, Reconciliation, Duplication, Loss, Synteny

## Abstract

The classical gene and species tree reconciliation, used to infer the history of gene gain and loss explaining the evolution of gene families, assumes an independent evolution for each family. While this assumption is reasonable for genes that are far apart in the genome, it is not appropriate for genes grouped into syntenic blocks, which are more plausibly the result of a concerted evolution. Here, we introduce the *Super-Reconciliation* problem which consists in inferring a history of segmental duplication and loss events (involving a set of neighboring genes) leading to a set of present-day syntenies from a single ancestral one. In other words, we extend the traditional Duplication-Loss reconciliation problem of a single gene tree, to a set of trees, accounting for segmental duplications and losses. Existency of a Super-Reconciliation depends on individual gene tree consistency. In addition, ignoring rearrangements implies that existency also depends on gene order consistency. We first show that the problem of reconstructing a most parsimonious Super-Reconciliation, if any, is NP-hard and give an exact exponential-time algorithm to solve it. Alternatively, we show that accounting for rearrangements in the evolutionary model, but still only minimizing segmental duplication and loss events, leads to an exact polynomial-time algorithm. We finally assess time efficiency of the former exponential time algorithm for the Duplication-Loss model on simulated datasets, and give a proof of concept on the opioid receptor genes.

## Background

Gene gain and loss is known as a major force driving evolution. The classical method used for inferring these events is to reconstruct the tree of the gene family of interest and to embed it into the species phylogeny. Assuming the gene and species trees are known and correspond to the true evolution, incongruence between the two trees can be explained by gain and loss events, and “reconciling” the two trees allows recovering these events.

Tree reconciliation can be performed through different biological models of evolution, the most common being the Duplication-Loss (DL) [1–3] or Duplication-Loss and Transfer [4–6] models. Incomplete lineage sorting, i.e. imperfect segregation of alleles, can also be considered [7, 8]. While most reconciliation methods are based on the parsimony principle of minimizing the number or cost of operations, probabilistic models seeking for a reconciliation with maximum likelihood or maximum posterior probability have also been developed [9–11].

Regardless of the model, current algorithms for reconciliation take each gene family individually, assuming an independent evolution through single gene gain and loss. Although this hypothesis is reasonable for genes that are far apart in the genome, it is clearly too restrictive for those organized in syntenic blocks or paralogons, i.e. sets of homologous chromosomal regions, among one or many genomes, sharing the same genes (e.g. neuropeptide Y-family receptors [12], the Homeobox gene clusters [13–15], the FGFR fibroblast growth factor receptors [16, 17] or the genes of the opioid system [18–20]). These genes are more plausibly the result of an evolution from a common ancestral region, rather than from a set of independent gene duplications that would have converged to the same organization in different genomic regions.

The purpose of this paper is to generalize the DL reconciliation of a single gene tree, to a set of gene trees, accounting for segmental duplications and losses. As far as we know, this problem has never been considered before. The closest algorithms are DeCo [21] and DeCoStar [22] which, given a set of gene families, a set of adjacencies between genes, a set of gene trees and a species tree, compute an adjacency forest reflecting the evolution of each adjacency. However, adjacencies are taken independently, and only single duplications and losses are considered. A correction strategy that adjusts the computation of the evolutionary cost to favour co-evolution events, hence grouping seemingly individual events into single segmental ones was latter proposed in [23]. Another related problem asks for the reconciliation of a set of gene trees leading to a minimum number of duplication episodes, referring to possible whole genome duplication events, defined as sets of single duplications mapped to the same node in the species tree [24, 25]. However the considered model does not account for gene orders and duplications involving a set of neighboring genes.

Here, we consider the *DL Super-Reconciliation* problem (or Super-Reconciliation for short when there is no ambiguity) in which, given a set of gene families, a set of syntenies (chromosomal segments exhibiting similar gene contents), a gene tree for each gene family and a species tree, we seek an evolutionary history of the set of syntenies that is in agreement with the individual gene trees whilst minimizing the number of segmental duplications and losses. Our proposed model is a direct generalization of the reconciliation of a single gene tree. Existency of a Super-Reconciliation depends on individual gene tree consistency. In addition, ignoring rearrangements implies that existency also depends on gene order consistency. We first show that the problem of reconstructing a most parsimonious Super-Reconciliation, if any, is NP-hard and give an exact exponential-time algorithm to solve it. Alternatively, we show that accounting for rearrangements in the evolutionary model, but still only minimizing segmental duplications and losss, reduces to ignoring gene orders in syntenies, and leads to an exact polynomial-time algorithm.

After defining the new Super-Reconciliation model in the next section, we characterize, in the “Existence conditions” section, the conditions under which a Super-Reconciliation exists for a set of syntenies and a set of gene trees, and exhibit a general framework for inferring a most parsimonious DL Super-Reconciliation. We prove, in the “Complexity of the Super-Reconciliation problem” section, that this problem is NP-hard. A dynamic programming algorithm for the main step of the framework is given in the “A Super-Reconciliation for a supertree” section. The “Unordered Super-Reconciliation” section is dedicated to an extension of the original evolutionary model accounting for rearrangements. We give a polynomial-time algorithm for finding a Super-Reconciliation, under this model, minimizing the number of segmental duplications and losses. An application on simulated datasets and a proof of concept on the genes of the opioid system are then presented in the “Application” section. We conclude with a discussion in the “Conclusion” section.

## Trees, reconciliation and problem statement

A *string* or a *sequence* is an ordered set of characters. Given a string $X = x_1 \cdots x_n$, a *substring* of *X* is a consecutive set of characters from *X* in the same order as in *X* (possibly *X* itself), and a *subsequence* is a set of characters of *X* in the same order, but not necessarily consecutive in *X* (*X* is a substring and a subsequence of *X*). We also denote by $Set(X) = \{x_1, x_2, ... , x_k\}$ the *range* of *X*, i.e. the set of all genes contained in *X*, without any particular order.

All trees are considered rooted. Given a tree *T*, we denote by *r*(*T*) its root, by *V*(*T*) its set of nodes and by ${{\mathcal {L}}}(T) \subseteq V(T)$ its leafset. We say that *T is a tree for*$L = {{\mathcal {L}}}(T)$. A node *v* is an *ancestor* of $v'$ if *v* is on the path from *r*(*T*) to $v'$; *v* is the *father* of $v'$ if it directly precedes $v'$ on this path. In this latter case, $v'$ is called the *child* of *v*. We denote by *E*(*T*) the set of edges of *T*, where an edge is represented by its two terminal nodes $(v,v')$, with *v* being the father of $v'$. Two nodes *v* and $v'$ are *separated* in *T* iff neither one is an ancestor of the other. A node is said to be *unary* if it has a single child and *binary* if it has two children. Given a node *v* of *T*, the subtree of *T* rooted at *v* is denoted *T*[*v*].

A *binary tree* is a tree with all internal (i.e. non-leaf) nodes being binary. If internal nodes have one or two children, then the tree is said *partially binary*.

*Creating a unary root* consists of creating a new node *v*, a new edge (*v*, *r*(*T*)) and assigning *v* as the new root of *T*. *Grafting* a leaf *w* consists of subdividing an edge $(v,v')$ of *T*, thereby creating a new node $v''$ between *v* and $v'$, then adding a leaf *w* with parent $v''$. If *W* is a rooted tree, *grafting W to T* corresponds to grafting a leaf *w*, then replacing *w* by the root of *W*.

The *lowest common ancestor* (LCA) in *T* of a subset $L'$ of ${{\mathcal {L}}}(T)$, denoted $lca_T(L^{\prime})$, is the ancestor common to all nodes in $L^{\prime}$ that is the most distant from the root. The restriction $T|_{L^{\prime}}$ of *T* to $L^{\prime}$ is the tree with leafset $L^{\prime}$ obtained from the subtree of *T* rooted at $lca_T(L^{\prime})$ by removing all leaves that are not in $L^{\prime}$ and all unary nodes. Let $T^{\prime}$ be a tree such that ${{\mathcal {L}}}(T') = L' \subseteq {{\mathcal {L}}}(T)$. We say that *Tdisplays*$T'$ iff $T|_{L'}$ is label-isomorphic to $T'$ (i.e., isomorphic with preservation of leaf labels). We also say that *T* is an *extension* of $T'$.

**Fig. 1:**
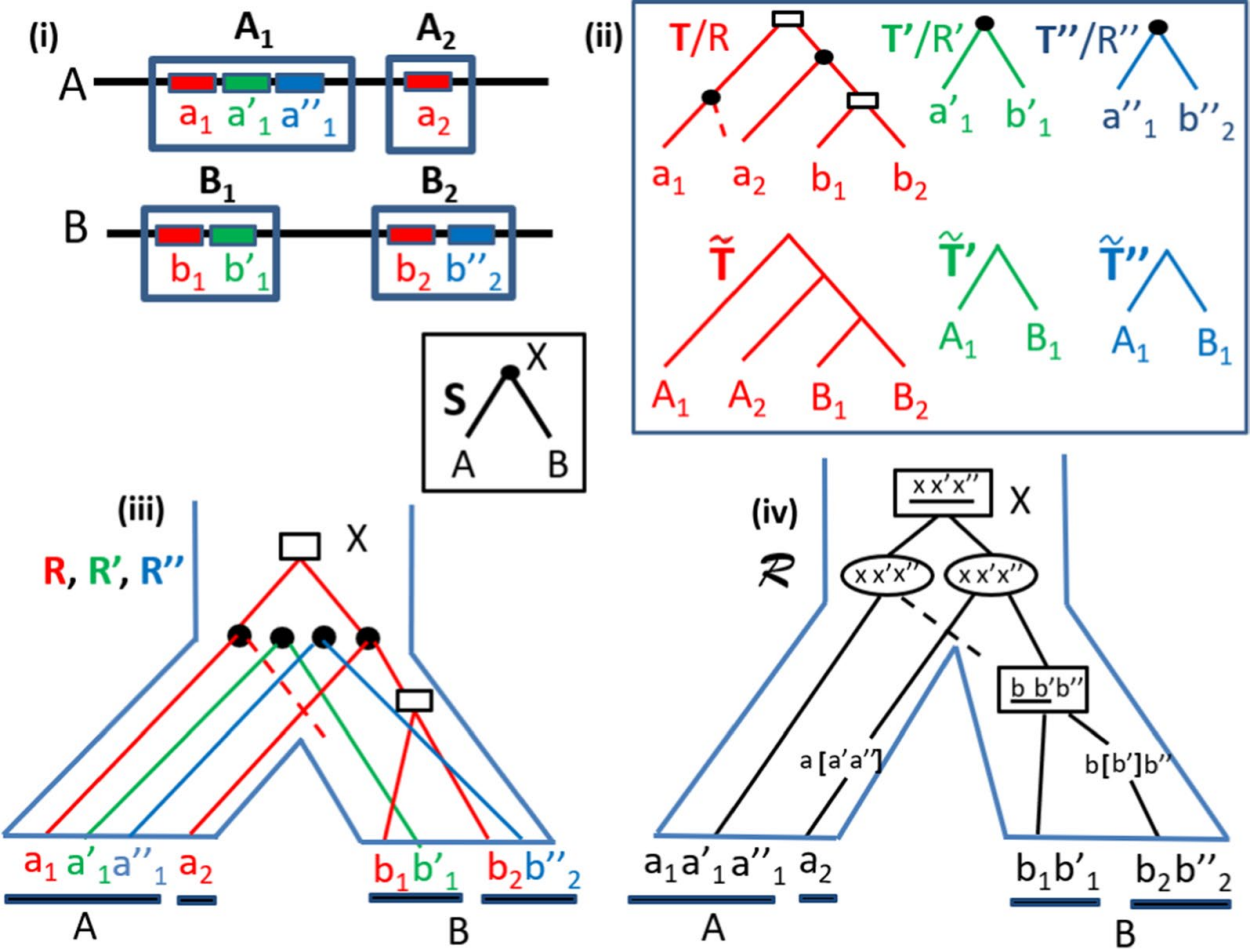
(i) Two genomes *A* and *B*; three gene families (red, green and blue) grouped into two syntenies $A_1, A_2$ in *A* and two syntenies $B_1, B_2$ in *B*. (ii) Ignoring node labels and dotted lines, *T*, $T'$ and $T''$ are the corresponding gene trees and $\tilde{T}$, $\tilde{T}'$ and $\tilde{T}''$ are the corresponding synteny trees. The reconciled gene trees *R*, $R'$ and $R''$ are the same trees but including node labels and dotted lines. Nodes identified by circles are speciations, those represented by rectangles are duplications, and dotted lines represent lost branches. (iii) The reconciled trees embedded into the species tree *S*. (iv) A Super-Reconciliation ${\mathcal {R}}$, representing a more realistic evolutionary history from a common ancestral synteny. Each ancestral node is identified by the synteny, the event and the segment of the synteny affected by the event. Square nodes represent *Dup* events, round nodes *Spe* events, brackets *pLoss* events and dotted lines *fLoss* (see text)

*Species, gene and synteny trees* (see Fig. 1) The *species treeS* for a set ${\Sigma }$ of species represents an ordered set of speciation events that have led to $\Sigma$.

A *gene family* is a set ${\Gamma }$ of genes where each gene *g* belongs to a given species *s*(*g*) of ${\Sigma }$. If $\Gamma ' \subseteq \Gamma$ is a subset of genes, we denote $s(\Gamma ') = \{s(g) : g \in \Gamma '\}$.

A *syntenyX* is an ordered sequence of genes belonging to a genome *s*(*X*). We consider that genes of a synteny all belong to different gene families (tandem duplications are ignored). More precisely, let ${\mathcal {F}}= \{{\Gamma }_1, {\Gamma }_2, ..., {\Gamma }_t\}$ be a set of gene families, and $\lambda _{\mathcal {F}}= \{(g, {\Gamma }) : g \in {\Gamma }\wedge {\Gamma }\in {\mathcal {F}}\}$ be a function. We say that an ordered sequence of genes $X = g_1 g_2 ... g_k$ is a synteny on ${\mathcal {F}}$ iff $\lambda _{\mathcal {F}}$ is well-defined for all genes of *X*, $\lambda _{\mathcal {F}}$ is injective, and all genes in *X* belong to the same species.

A *synteny family* is a set ${{\mathcal {X}}}$ of syntenies. We say that a set ${\mathcal {F}}$ of gene families are *organized into a set*${{\mathcal {X}}}$*of syntenies* iff there is a bijection between the genes of ${\mathcal {F}}$ and the genes in ${{\mathcal {X}}}$ (each gene of ${\mathcal {F}}$ belongs to exactly one synteny of ${{\mathcal {X}}}$).

A tree *T* is a *gene tree* for a gene family ${\Gamma }$ (respec. a *synteny tree* for a synteny family ${{\mathcal {X}}}$) if its leafset is in bijection with ${\Gamma }$ (respec. ${{\mathcal {X}}}$).

Given a gene tree *T*, the *corresponding synteny tree* is the tree $\tilde{T}$ obtained from *T* by replacing each leaf of *T* by the synteny containing the considered gene.

Given a tree *T* (either gene tree or synteny tree), we extend the mapping *s* to internal nodes *v* of *T* by defining $s(v) = lca_S( \{s(l) : l \in {{\mathcal {L}}}(T[v]) \} )$.

An evolutionary history is represented by a *labeled* tree, where the label of a node is its corresponding event. In the case of gene families, an event is entirely determined by its type, either a duplication, a speciation or a loss. The labels of a gene tree are obtained through reconciliation, as described below.

### Reconciliation

#### **Definition 1**

(Reconciled gene tree) Let *T* be a binary gene tree and *S* be a binary species tree. A *DL Reconciliation* (or simply *reconciliation*) *R*(*T*, *S*) of *T* with *S* is a labeled extension of *T* obtained by grafting new leaves satisfying: for each internal node *v* of *R*(*T*, *S*) with two children $v_l$ and $v_r$, either $s(v_l) = s(v_r) = s(v)$, or $s(v_l)$ and $s(v_r)$ are the two children of *s*(*v*). The node *v* is a duplication in *s*(*v*) in the former case and a speciation in the latter case. A grafted leaf on a newly created node *v* corresponds to a loss in *s*(*v*). All other leaves are labeled by the default event “extant”.

The cost of a reconciliation *R*(*T*, *S*) is the number of induced duplications and losses.

Note that in a reconciliation, we only choose *s*(*l*) for the grafted leaves, and the value of *s*(*v*) for the internal nodes is entirely determined by the leaves descending *v*. Given a gene tree *T* and a species tree *S*, a *minimum reconciliation*, i.e. a reconciliation of minimum cost, is obtained from the LCA-mapping which consists in setting $s(v) = lca_S(s({{\mathcal {L}}}(T[v])))$ for each $v \in V(T)$, and labeling each internal node *v* of *T* as a speciation if and only if $s(v_l)$ and $s(v_r)$ are separated in *S*, and as a duplication otherwise. Observe that in any case, if $s(v_l)$ and $s(v_r)$ are not separated, then it is impossible for *v* to be a speciation. We denote by *LCA-Reconciliation* the reconciliation labeled by means of the LCA-mapping.

Before extending the reconciliation concept to a set of gene trees, we need to specify an evolutionary model for syntenies. In this paper, syntenies are considered to have evolved from a single ancestral synteny through speciations (defined as for single genes), segmental duplications and segmental losses, where:

- A speciation *Spe*(*X*, [1, *l*]) acting on a synteny $X = g_1 \cdots g_l$ belonging to a genome *s*(*X*) has the effect of reproducing *X* in the two genomes $s_l$ and $s_r$ children of *s*(*X*) in *S*.
- A (segmental) duplication *Dup*(*X*, [*i*, *j*]) acting on a synteny *X* belonging to a genome *s*(*X*) is an operation that copies a substring $g_i \cdots g_j$ of size $j-i+1$ of $X=g_1 g_2 \cdots g_i \cdots g_j \cdots g_l$ somewhere else into the genome *s*(*X*), creating a new *copied synteny*$X' = g'_i \cdots g'_j$ where each $g'_k$, for $i \le k \le j$ belongs to the same gene family as $g_k$; we say that the copied synteny is *partial* if $[i,j] \ne [1,l]$.
- A (segmental) loss *Loss*(*X*, [*i*, *j*]) acting on a synteny $X=g_1 \cdots g_i \cdots g_j \cdots g_l$ is an operation that removes a substring $g_i \cdots g_j$ of size $j-i+1$ of *X*, leading to the *truncated synteny*$X' = g_1 \cdots g_{i-1} g_{j+1} \cdots g_l$. A loss is called *full* if $X'$ is the empty string (i.e. all genes of *X* are removed) and *partial* otherwise. We may a denote full loss event as *fLoss* and a partial loss event as *pLoss*.

An evolutionary history of a set of syntenies can thus be represented as a partially binary tree where leaves correspond to extant syntenies and lost syntenies (resulting from full losses), and each internal node *v* corresponds to an event ${\mathcal {E}}(X, [i,j])$ with ${\mathcal {E}} \in \{Spe, Dup, pLoss\}$. Thus, in contrast to a single gene family, a tree representing the evolution of a set of syntenies is not only labeled by the type of event corresponding to each internal node, but also by the segment of the synteny affected by the event (see the bottom-right tree in Fig. 1).

If $${\mathcal {E}}$$ is:

1. *Spe*, then *v* is a binary node with two children corresponding to syntenies *Y* and *Z* such that $X= Y = Z$ and *s*(*Y*) and *s*(*Z*) being the two children of *s*(*X*) in *S*.
2. *Dup*, then *v* is a binary node with two children corresponding to syntenies *X* and $X'=X[i,j]$, where $s(X) = s(X')$.
3. *pLoss*, then *v* is a unary node with a child corresponding to the truncated synteny $X'=X[1,i-1]X[j+1,l]$, and $s(X) = s(X')$.

The topology of a tree representing the evolution of a set of syntenies differs from that of a single gene family since the former may contain unary nodes, resulting from partial losses, while the latter only contains binary nodes.

Our goal is to infer an evolutionary history of a set of syntenies which is a reconciliation of a set of individual gene trees, formally defined below.

#### **Definition 2**

(Super-Reconciliation) Let ${\mathcal {G}}= \{T_1, T_2, \cdots , T_t\}$ be a set of binary gene trees for the gene families ${\mathcal {F}}= \{{\Gamma }_1, {\Gamma }_2, \cdots , {\Gamma }_t\}$ organized into a set ${{\mathcal {X}}}$ of syntenies belonging to a set ${\Sigma }$ of taxa, and let *S* be a binary species tree for ${\Sigma }$. For each *i*, $1 \le i \le t$, let $\tilde{T}_i$ be the synteny tree corresponding to $T_i$.

A *Super-Reconciliation*$R({\mathcal {G}},S)$ of ${\mathcal {G}}$ with *S* is a labeled synteny tree which is an extension of the trees $\tilde{T}_i$, for $1 \le i \le t$, representing a valid history for ${{\mathcal {X}}}$.

The cost of a Super-Reconciliation $R({\mathcal {G}},S)$ is the number of induced *Dup*, *fLoss* and *pLoss* events.

For example, the cost of the Super-Reconciliation in Fig. 1 is 5. Notice that, although this cost is higher than that obtained by considering each gene family independently (cost of 3), the induced history is much more realistic as it is unlikely that independent gene duplications would have led to the same gene organization in different genomic regions.

We are now ready to state the optimization problem considered in this paper.

super-reconciliation problem:

**Input:** A set ${\Sigma }$ of species and a species tree *S* for ${\Sigma }$; a set of gene families ${\mathcal {F}}= \{{\Gamma }_1, {\Gamma }_2, \cdots , {\Gamma }_t\}$ organized into a set of syntenies ${{\mathcal {X}}}$; a set of gene trees ${\mathcal {G}}= \{T_1, T_2 \cdots , T_t\}$ one for each family of ${\mathcal {F}}$.

**Output:** A Super-Reconciliation $R({\mathcal {G}},S)$ of minimum cost.

## Existence conditions

As a synteny is represented by a gene order and can only be modified through losses (duplications create new syntenies but do not modify existing syntenies), an evolutionary history does not always exist for a set of syntenies ${{\mathcal {X}}}$, regardless of the trees linking them. If this holds, the syntenies are said to be *order consistent*.

In addition, in contrast to the reconciliation of a single gene tree which always exists, this is not the case for a Super-Reconciliation as different gene trees may exhibit inconsistent speciation histories for the same syntenies.

The following two subsections are dedicated to characterizing the gene order and gene tree conditions required for the existence of a Super-Reconciliation.

### Consistency of gene orders

Given a set of gene families ${\mathcal {F}}= \{{\Gamma }_1, {\Gamma }_2, \cdots , {\Gamma }_t\}$ organized into a set of syntenies ${{\mathcal {X}}}$, we define the *precedence graph*${\mathcal {P}}$ as the directed graph with *n* vertices, each corresponding to a gene family of ${\mathcal {F}}$, such that a directed edge (*i*, *j*) between two vertices *i* and *j* exists iff there is a synteny $X=x_1 x_2 \cdots x_k$ of ${{\mathcal {X}}}$ containing a gene in ${\Gamma }_i$ preceding a gene in ${\Gamma }_j$, i.e. there is a pair $1 \le l_1 < l_2 \le k$ such that $x_{l_1} \in {\Gamma }_i$ and $x_{l_2} \in {\Gamma }_j$.

If ${\mathcal {P}}$ is acyclic, then ${\mathcal {P}}$ is a Directed Acyclic Graph (DAG). In this case, there is a topological sorting for ${\mathcal {P}}$, i.e., a linear ordering *X* of vertices such that for every directed edge (*i*, *j*) in ${\mathcal {P}}$, *i* precedes *j* in *X*. Verifying if a directed graph is acyclic and finding a topological sorting of a DAG is a classical problem solvable in linear time.

The following lemma gives necessary and sufficient conditions for a set of syntenies to be order consistent and exhibits the set of possible ancestral syntenies.

#### **Lemma 1**

(Order consistency condition) *Let*${\mathcal {F}}= \{{\Gamma }_1, {\Gamma }_2, \cdots , {\Gamma }_t\}$*be a set of gene families organized into a set*${{\mathcal {X}}}$*of syntenies. Then*${{\mathcal {X}}}$*is order consistent iff the corresponding precedence graph*${\mathcal {P}}$*is acyclic. In this case, any topological sorting for*${\mathcal {P}}$*is an order consistent ancestral synteny for*${{\mathcal {X}}}$.

#### *Proof*

The first part of the lemma follows from the fact that a directed graph has a topological sorting if and only if it is acyclic. The second part follows from the fact that, for any topological sorting *A* for ${\mathcal {P}}$ and any synteny *X* of ${{\mathcal {X}}}$, *X* is a subsequence of *A*, and thus *X* can be obtained from *A* through losses. $\square$

The ancestral synteny *A* at the root of a Super-Reconciliation $R({\mathcal {G}},S)$ is an order on ${\mathcal {F}}$. Moreover, as the synteny at each internal node of $R({\mathcal {G}},S)$ is obtained from *A* through losses, a synteny at each internal node of $R({\mathcal {G}},S)$ should be a subsequence of *A*. More generally, for any two nodes *v* and $v'$ of $R({\mathcal {G}},S)$, where *v* is an ancestor of $v'$, the synteny *Y* at $v'$ is a subsequence of the synteny *X* at *v*.

### Consistency of trees

A set of trees on subsets of ${{\mathcal {X}}}$ is said *consistent* iff, for any triplet $Trp=\{X_1, X_2, X_3\}$ of distinct elements of ${{\mathcal {X}}}$, all trees containing *Trp* as a sub-leafset exhibit the same topology for *Trp*.

#### **Lemma 2**

(Tree consistency condition) *Let*${\mathcal {G}}= \{T_1, T_2, \cdots , T_t\}$*be a set of gene trees for a set of gene families organized into a set*${{\mathcal {X}}}$*of syntenies, and letSbe the species tree. If a Super-Reconciliation*$R({\mathcal {G}},S)$*exists, then the set of corresponding synteny trees*$\{\tilde{T}_1, \tilde{T}_2, \cdots \tilde{T}_t\}$*is consistent.*

#### *Proof*

By definition, a Super-Reconciliation $R({\mathcal {G}},S)$ displays $\tilde{T}_i$, for all $1 \le i \le t$, as $R({\mathcal {G}}, S)$ is an extension of each tree. Thus, for any triplet $Trp=\{X_1, X_2, X_3\}$ of ${{\mathcal {X}}}$, if $\tilde{T}_i$ and $\tilde{T}_j$ contain the triplet *Trp* as a sub-leafset, then $R({\mathcal {G}},S)$ displays both $\tilde{T}_i|_{Trp}$ and $\tilde{T}_j|_{Trp}$. In other words, $\tilde{T}_i|_{Trp}$ and $\tilde{T}_j|_{Trp}$ are label-isomorphic. $\square$

The consistency problem of rooted trees has been widely studied. The BUILD algorithm [26] can be used to test, in polynomial-time, whether a collection of rooted trees is consistent, and if so, construct a compatible, not necessarily fully resolved, supertree, i.e. a tree displaying them all. This algorithm has been generalized to output all compatible minimally resolved supertrees [27–29], which may be exponential in the number of genes.

The following theorem makes the link between a supertree and a reconciliation.

#### **Theorem 1**

*Let*${\mathcal {G}}= \{T_1, T_2 \cdots , T_t\}$*be a set of trees for a set of families organized in an order-consistent set of syntenies*${{\mathcal {X}}}$, *andSbe the species tree. Let*$\tilde{{\mathcal {G}}} = \{\tilde{T}_1, \tilde{T}_2 \cdots , \tilde{T}_t\}$*be the set of synteny trees corresponding to those in*${\mathcal {G}}$. *If*$\tilde{{\mathcal {G}}}$*is a consistent set of trees then:*

1. *A Super-Reconciliation*$R({\mathcal {G}},S)$*is an extension of a supertree for*$\tilde{{\mathcal {G}}}$;
2. *Any supertree is the “backbone” of a Super-Reconciliation. Namely, for any supertree*$\tilde{T}$*for*$\tilde{{\mathcal {G}}}$, *there is a Super-Reconciliation*$R({\mathcal {G}},S)$*which is an extension of*$\tilde{T}$.

The first statement of Theorem 1 follows from Lemma 2. As for the second statement, we will prove it implicitly in the “A Super-Reconciliation for a supertree” section by providing an algorithm that yields a minimum cost reconciliation on any supertree.

Following Theorem 1, the problem reduces to finding a supertree for the set of synteny trees minimizing the number of segmental duplications and losses. A natural algorithm for the super-reconciliation problem follows:

1. Explore the space of all order consistent ancestral syntenies *A* for ${{\mathcal {X}}}$;
2. Explore the space of all supertrees $\tilde{T}$ for $\tilde{{\mathcal {G}}}$;
3. Find a Super-Reconciliation of minimum cost which is an extension of $\tilde{T}$ with *A* as an ancestral synteny;
4. Select the Super-Reconciliations leading to the minimum cost.

Step 1 and Step 2 have been discussed in this section. Before developing an algorithm for Step 3, which is the purpose of the “A Super-Reconciliation for a supertree” section, we begin by analyzing the theoretical complexity of the super-reconciliation problem.

## Complexity of the Super-Reconciliation problem

We have recently considered the problem of finding a supertree of a set of gene trees minimizing the classical single gene duplication and single gene duplication and loss distances. The problem has been shown NP-hard for the duplication distance, and exponential-time algorithms have been developed for both distances.

For segmental duplications only, the hardness of super-reconciliation is almost immediate from the results of [30]. For both duplications and losses, the problem remains NP-hard, although the proof is far more technical. Here we give the simpler proof of hardness for minimizing duplications only, and refer the reader to Additional file 1 for the NP-hardness proof for minimizing segmental duplications *and* losses.

### **Theorem 2**

*The*super-reconciliation*problem is NP-hard for the duplication cost. Furthermore, the minimum number of duplications is hard to approximate within a factor*$n^{1 - \epsilon }$*for any*$0< \epsilon < 1$, *wherenis the number of syntenies in the input.*

### *Proof*

The hardness follows from that of the mindup-supertree problem, defined as follows. Given a species tree *S* and a set of gene trees $T_1, \ldots , T_k$, possibly with overlapping leafsets, mindup-supertree asks for a supertree *T* that displays $T_1, \ldots , T_k$ such that the LCA-reconciliation of *T* and *S* yields a minimum number *d* of duplications. It was shown in [30] that it is NP-hard to approximate *d* within a factor $n^{1 - \epsilon }$ for any $0< \epsilon < 1$, where here *n* is the number of genes in $\Gamma = \bigcup _{i = 1}^k {{\mathcal {L}}}(T_i)$.

To reduce mindup-supertree to the super-reconciliation problem, it essentially suffices to exchange the roles of genes and syntenies. More precisely, given an instance of mindup-supertree consisting of a species tree *S* and gene trees $T_1, \ldots , T_k$, we compute an instance of super-reconciliation as follows. The species tree is the same as *S*, and for each gene $g \in \Gamma$, we have a synteny $X_g$ with $s(X_g) = s(g)$ whose gene content will soon be defined. For each gene tree $T_i$, we create an identical gene tree $T'_i$, but in which each gene $g \in {{\mathcal {L}}}(T_i)$ is replaced by a unique gene $g_{T_i}$ that belongs to synteny $X_g$ (and hence $s(g) = s(g_{T_i}) = s(X_g)$). Thus $X_g$ has one gene $g_{T_i}$ for each occurrence of *g* in a tree $T_i$ (recall that a gene can occur in multiple trees). The ordering of the genes of $X_g$ is arbitrary (since we are not counting segmental losses), but the ordering must be order-consistent. This is easily achieved by ordering the $g_{T_i}$’s of each synteny $X_g$ in ascending order of their *i* indices. Note that the synteny tree $\tilde{T}_i$ for $T'_i$ is obtained by replacing each leaf *g* of $T_i$ by $X_g$. Also observe that there are *n* syntenies.

It only remains to show the correspondence between the solutions for the two problem instances. Suppose that the mindup-supertree instance admits a supertree *T* with *d* duplications when reconciled. Let $\tilde{T}$ be the synteny tree obtained from *T* by replacing each gene $g \in {{\mathcal {L}}}(T)$ by $X_g$. Because $s(g) = s(X_g)$, both *T* and $\tilde{T}$ have the same duplications under the LCA-reconciliation, which is *d*. One can assign a synteny *X* at the root of $\tilde{T}$ that satisfies our constraints defined by the precedence graph. We assign the same synteny *X* at every internal node (so no partial losses or partial duplications), and partial losses can be added on each edge linking a leaf $X_g$ with its parent to obtain the $X_g$ synteny. As we are not counting losses, these are irrelevant and we achieve a cost of *d* duplications. Conversely, assume that the super-reconciliation instance formed by $T'_1, \ldots , T'_k$ and *S* admits a synteny tree $\tilde{T}$ with *d* duplications. We may replace each leaf $X_g$ by *g*, yielding an extension of a supertree *T* for the mindup-supertree instance. After suppressing unary vertices, this results in a reconciled gene supertree with *d* duplications. Because the value of the solutions are preserved and $n = |\Gamma |$ corresponds to the number of syntenies, this reduction is approximation-preserving and the hardness result follows. $\square$

We state our second hardness result formally here.

### **Theorem 3**

*The*super-reconciliation*problem is NP-hard for theDup*, *fLossandpLosscost.*

## A Super-Reconciliation for a supertree

In this section, we are given a set ${\mathcal {G}}= \{T_1, T_2, \cdots , T_t\}$ of consistent gene trees for a set of families ${\mathcal {F}}= \{{\Gamma }_1, {\Gamma }_2, \cdots , {\Gamma }_t\}$ organized in an order consistent set of syntenies ${{\mathcal {X}}}$, and a species tree *S* for the set ${\Sigma }$ of taxa containing the genes. In addition, we are given a supertree $\tilde{T}$ for the synteny trees $\tilde{{\mathcal {G}}} = \{\tilde{T}_1, \tilde{T}_2, \cdots , \tilde{T}_t\}$ corresponding to those in ${\mathcal {G}}$, and an order consistent ancestral synteny *A* for ${{\mathcal {X}}}$.

Given a Super-Reconciliation $R({\mathcal {G}},S)$ (*R* for short), because *R* is obtained from $\tilde{T}$ by grafting leaves, each node of $\tilde{T}$ is present in *R*. Hence we say that $v \in V(\tilde{T})$ has a *corresponding node*$v'$ in *R*. More precisely, if $l \in {{\mathcal {L}}}(\tilde{T})$, then $l \in {{\mathcal {L}}}(R)$ also and the correspondence is immediate. If *v* is an internal node of $V(\tilde{T})$, the node $v'$ of *R* corresponding to *v* is $lca_R( \{l : l \in {{\mathcal {L}}}(\tilde{T}[v]) \} )$. We show that, as in the traditional reconciliation setting, the nodes of *R* that are also in $\tilde{T}$ should be mapped to the lowest species possible. To simplify the argument, we will call an internal node a full loss if it is the parent of a *fLoss* event. For later reference, we note that the proofs of Lemma 3 and Lemma 4 do not involve the gene orders in any way.

### **Lemma 3**

*Let*$R({\mathcal {G}},S)$*be a Super-Reconciliation of minimum cost which is an extension of*$\tilde{T}$. *Let*$v \in V(\tilde{T})$*and let*$v'$*be the node corresponding tovin*$R({\mathcal {G}}, S)$. *Then*$s(v') = lca_S( s({{\mathcal {L}}}(\tilde{T}[v]) ) )$.

### *Proof*

First, observe that the statement is clearly true for the leaves. Assume that the statement is false. Now, let *v* be a node of $\tilde{T}$ such that its corresponding node $v'$ does not satisfy the statement—moreover, choose *v* to be a minimal node with this property (meaning that for the children $v_l$ and $v_r$ of *v*, the corresponding nodes $v_l'$ and $v_r'$ in $R({\mathcal {G}}, S)$ satisfy $s(v_l') = lca_S( s({{\mathcal {L}}}(\tilde{T}[v_l]) )$ and $s(v_r') = lca_S( s({{\mathcal {L}}}(\tilde{T}[v_r]) )$). Note that *v* must exist, since the statement is true for the leaves.

Now, we may assume that $s(v') \ne lca_S(s(v_l'), s(v_r'))$, as otherwise $v'$ satisfies the lemma. Thus in *S*, there are at least *k* edges on the path from $s(v')$ to $lca_S(s(v_l'), s(v_r'))$, where here $k > 0$. It is not hard to verify that in this case, $v'$ must be a duplication node, according to the definition of a reconciliation. This implies that there are at least *k* full losses on the path from $v'$ to $v_l'$ and at least *k* full losses on the path from $v'$ to $v_r'$. Consider the Super-Reconciliation $R'$ that is identical to $R({\mathcal {G}}, S)$, with the exception that $s(v')=lca_S(s(v_l'), s(v_r'))$. Then the 2*k* losses on the paths between $v'$ and $v_l'$ and between $v'$ and $v_r'$ are not needed anymore, although if $v'$ is not the root, *k* losses become necessary on the path between $v'$ and $w'$, where $w'$ is the node corresponding to the parent *w* of *v* in $\tilde{T}$. Remapping $v'$ cannot increase the number of duplications, and so we have saved *k* losses.

It remains to argue that the number of partial losses remains the same. But this is easy to see. We keep the same synteny assignment at nodes $v'$, $v'_l$ and $v'_r$ (and $w'$ if $v'$ is not the root) as in $R({\mathcal {G}}, S)$. If $v'$ was a segmental duplication in $R({\mathcal {G}}, S)$, we set $v'$ to be a segmental duplication in $R'$ as well. The number of partial losses on the paths between $v'$ and $v_l', v_r'$ (and $w'$) therefore remains the same as in $R({\mathcal {G}}, S)$. $\square$

We now show that speciation and duplication nodes are easy to identify. Essentially, we may set the events of internal nodes as in the classical LCA-mapping reconciliation. In what follows, assume that $\tilde{T}$ is reconciled under the LCA-mapping, and put $s(v) = lca_S({{\mathcal {L}}}(s(\tilde{T}[v])))$ for every $v \in V(\tilde{T})$.

### **Lemma 4**

*Let*$R({\mathcal {G}},S)$*be a Super-Reconciliation of minimum cost which is an extension of*$\tilde{T}$. *Let*$v \in V(\tilde{T})$*be an internal node of*$\tilde{T}$*and let*$v'$*be its corresponding node in*$R({\mathcal {G}},S)$. *Moreover let*$v_l$*and*$v_r$*be the children ofv*. *If*$s(v_l)$*and*$s(v_r)$*are separated inS*, *then*$v'$*is a speciation, and otherwise*$v'$*is a duplication.*

### *Proof*

Let $v_l'$ and $v_r'$ be the nodes corresponding to $v_l$ and $v_r$, respectively, in $R({\mathcal {G}}, S)$. First, if $s(v_l)$ and $s(v_r)$ are not separated, then by Lemma 3, $s(v_l')$ and $s(v_r')$ are not separated, hence it is not possible for $v'$ to be a speciation. Therefore $v'$ must be a duplication.

Suppose instead that $s(v_l)$ and $s(v_r)$ are separated in *S*, but that $v'$ is labeled by a duplication event *Dup*(*X*, [*i*, *j*]), where *X* is the synteny assigned at $v'$. On the path from $v'$ to $v'_l$, there may be some *pLoss* events and some nodes that were grafted owing to full losses. We may assume that all full loss events, if any, have occurred before the *pLoss* events on this path (i.e., nodes grafted from full losses are closer to $v'$). This is without loss of generality, as this does not change the resulting synteny in $v'_l$. We shall make the same assumption with the path from $v'$ to $v'_r$. Now, by Lemma 3, $s(v') = lca_S(s(v_l), s(v_r))$. Because $v'$ is a duplication, the two children $w_l, w_r$ of $v'$ in $R({\mathcal {G}}, S)$ must satisfy $s(w_l) = s(w_r) = s(v')$. Since $s(v_l') \ne s(v') \ne s(v'_r)$, we have that $\{w_l, w_r\} \cap \{v_l', v_r'\} = \emptyset$, and therefore $w_l$ and $w_r$ were grafted on $\tilde{T}$ due to full losses. If we label $v'$ as a speciation *Spe*(*X*, [1, |*X*|]), these two full losses are not needed anymore, and by doing so we have one duplication less and two full losses less. Let $Y_l$ and $Y_r$ be the two syntenies that are assigned at $w_l$ and $w_r$ in $R({\mathcal {G}}, S)$, respectively. Then $Y_l = X$ and $Y_r = X[i,j]$ or vice-versa (assume the former, without loss of generality). Suppose that $w_r$ is an ancestor of $v'_r$ in $R({\mathcal {G}}, S)$, again without loss of generality. The substring *X*[*i*, *j*] can be obtained from *X* by adding at most two partial losses on the path from $v'$ to $v'_r$. The rest of the reconciliation can remain the same. To sum up, we have removed one duplication and two full losses, and inserted at most two partial losses to reproduce the effect of the segmental duplication. This contradicts that $R({\mathcal {G}}, S)$ is a reconciliation of minimum cost. $\square$

From Lemma 4, it follows that we know the event-type (Dup or Spe) of each internal node of the supertree $\tilde{T}$. It then remains to extend the tree with losses and infer the actual event at each node (i.e., the corresponding synteny and segment being duplicated or lost). It is easy to see that losses and segments affected by the events are fully determined by gene orders assigned to internal nodes. Therefore, the problem reduces to the classical “small phylogeny problem” generally defined as follows: Given an alphabet $\Sigma$ (nucleotides or amino-acids or genes), a distance on the set of words of $\Sigma$ (edit distance for gene sequences or rearrangement distances for gene orders) and a tree *T* with leaves being words on $\Sigma$ (extant gene sequences or gene orders), find the labeling of ancestral nodes (ancestral sequences or orders) minimizing the total cost of the tree. This cost is the sum of costs of each branch, which is the distance between the two words connected by the branch.

Here, we are given a synteny tree $\tilde{T}$ for a set ${{\mathcal {X}}}$ of syntenies on a set of gene families ${\mathcal {F}}$, and an ancestral synteny *A* which is an order of ${\mathcal {F}}$. We want to find a *synteny assignment* attributing a partial order on ${\mathcal {F}}$ to each node of $V(\tilde{T})$. We assume that the root *r* of $\tilde{T}$ is assigned the synteny *A*. It follows from the considered evolutionary model that, for two nodes *u* and *v* of $\tilde{T}$ with *u* being an ancestor of *v*, the synteny $X_v$ assigned to *v* should be a subsequence of the string $X_u$ assigned to *u*. A synteny assignment verifying this condition is called a *valid synteny assignment* for $\tilde{T}$.

For $v \in V(\tilde{T})$, define *d*(*v*, *X*) as the minimum number of segmental duplications and losses induced by a synteny assignment on $\tilde{T}[v]$ with *X* being the assignment at *v*. The small-phylogeny for syntenies problem is to find an optimal assignment, i.e. an assignment leading to $d(\tilde{T})= \min _X d(r(\tilde{T}),X)$ for *X* belonging to the set of syntenies that are order consistent with ${{\mathcal {X}}}$.

Solving this problem can be done by dynamic programming by computing *d*(*v*, *X*), for each $v \in V(\tilde{T})$ and each possible synteny *X*.

Let *v* be an internal node of $\tilde{T}$ and $v_l$, $v_r$ be its two children. Let *X*, $X_l$, $X_r$ be valid assignments for respectively *v*, $v_l$ and $v_r$. Then $X_l$ and $X_r$ are subsequences of *X*. If *v* is a speciation, then all missing genes in $X_l$ and $X_r$ are the result of losses. Otherwise, if *v* is a duplication, then for at most one of $X_l$ and $X_r$, the missing prefix or suffix can be due to the partial duplication of a segment of *X*, and all other missing genes should be the result of losses. This motivates the following two variants of the loss distance between two syntenies.

Let *X* and *Y* be two syntenies with *Y* being a subsequence of *X*. We let $D^{T}(X,Y)$ denote the minimum number of segmental losses required to transform *X* to *Y* and $D^{P}(X,Y)$ the minimum number of segmental losses required to transform a substring of *X* to *Y*.

### **Theorem 4**

*Letvbe a node of*$\tilde{T}$, *Xbe a synteny and*$\mathcal {S}(X)$*be the set of subsequences ofX*.

- *Ifvis a leaf, then*$d(v,X)=0$*ifXis the extant synteny corresponding to leafv*, *and*$+\infty$*otherwise;*
- *Ifvis a speciation with children*$v_l$*and*$v_r$, *then,*
  $$\begin{aligned} d(v,X)&= \text{ min }_{(X_l \in \mathcal {S}(X))} {(D^T(X, X_l) +d(v_l,X_l))} \\&\quad +\text{ min }_{(X_r\in \mathcal {S}(X))}{(D^T(X, X_r) +d(v_r,X_r))}; \end{aligned}$$
- *Ifvis a duplication node with children*$v_l$*and*$v_r$, *then*
  $$\begin{aligned} d(v,X)= 1 + \\\text{ min } {\left\{ \begin{array}{ll} \text{ min }_{(X_l\in \mathcal {S}(X))} (D^T(X,X_l) + d(v_l,X_l))+ \\ \text{ min }_{(X_r\in \mathcal {S}(X))} (D^P(X,X_r) + d(v_r,X_r)),\\ \text{ min }_{(X_l\in \mathcal {S}(X))} (D^P(X,X_l) + d(v_l,X_l))+ \\ \text{ min }_{(X_r\in \mathcal {S}(X))} (D^T(X,X_r) + d(v_r,X_r))\\ \end{array}\right. } \end{aligned}$$

The above can be used to solve the small-phylogeny for syntenies problem with dynamic programming. To do this, one can simply traverse $\tilde{T}$ in post-order, and apply the recurrences of Theorem 4 at each node encountered. We finish this section by analyzing the complexity of this algorithm. Let $n = |V(\tilde{T})|$ and let *t* be the number of gene families involved in the small-phylogeny for syntenies problem instance. For a node $v \in V(\tilde{T})$ and a synteny *X*, there are $O(2^t)$ possible subsequences of *X*. The value of *d*(*v*, *X*) can thus be computed by finding a minimum over $O(2^t)$ possible values for its left child $v_l$, and then over $O(2^t)$ possible values for its right child $v_r$. It is straightforward to check that $D^T$ and $D^P$ can be computed in time *O*(*t*) since all characters of the synteny strings are unique.

Let us now consider the number of possible entries in our dynamic programming table. The possible syntenies for *X* correspond to the subsequences of a topological sorting of an acyclic directed graph with *t* nodes (see Additional file 1). In the worst case, there are $O(2^t \cdot t!) = O(2^{t\log t + t})$ such syntenies. It follows that there are at most $O(n 2^{t \log t + t})$ entries in the dynamic programming table, and each entry takes time $O(t2^t)$ to compute. It is known that if there are *k* possible topological sortings in a directed acyclic graph, then they can be enumerated in time *O*(*k*) [31] (it is worth noting however that counting the number of such topological sortings in #P-complete [32]). Therefore, if *t* is not too large, then the above recurrences can solve the small phylogeny problem relatively quickly, even if *n* is large. Put differently, the small-phylogeny for syntenies problem is *fixed-parameter tractable* with respect to parameter *t*.

### **Corollary 1**

*The*small-phylogeny for syntenies*problem can be solved in time*$O(t2^{t \log t + 2t} n)$, *wheretis the number of gene families present in the input andnis the number of syntenies.*

## Unordered Super-Reconciliation

The strongest and less biologically supported condition for the existency of a DL Super-Reconciliation is probably gene order consistency. In fact, genomes being subject to rearrangements shuffling gene organization, it is hard to expect that a set of homologous chromosomal segments in phylogenetically distant genomes would exhibit the same gene order. In other words, we can hardly ignore the presence of rearrangements in the evolutionary history leading to a set of homologous genomic regions.

The small phylogeny problem, which consists in inferring ancestral gene orders minimizing a given rearrangement distance, has been extensively studied (see for example [33–37]). Algorithmic developments and results differ depending on the considered rearrangement distance. The most studied one is probably the DCJ distance, accounting for artificial movements implicitely mimicking inversions and transpositions [38, 39].

Almost all versions of the small phylogeny problem with rearrangements have been proven NP-hard, even those accounting for equal gene content for all genomes [40]. Heuristics have also been developed for inferring ancestral gene orders minimizing rearrangements, duplications and loss events (reviews can be found in [41, 42]). Extension of these heuristics to the Super-Reconciliation problem is certainly possible, but can only increase the intractability of the original problem.

Here, we explore a compromise which consists in considering an evolutionary model accounting for segmental duplications, losses and rearrangements, but yet only minimizing duplication and loss events. In other words, gene orders are not important anymore, as we can use as many rearrangements as we want for obtaining the required orders.

Reducing syntenies to their range sets, an *unordered evolutionary history* of a set of syntenies can be represented as a partially binary tree where each internal node *v* corresponds to an event ${\mathcal {E}}({\mathrm {Set}}(X))$ with $X= synteny(v)$ being the synteny at *v* and ${\mathcal {E}} \in \{Spe, Dup, pLoss\}$ such that, if ${\mathcal {E}}$ is:

1. *Spe*, then *v* is a binary node with two children corresponding to syntenies *Y* and *Z* such that ${\mathrm {Set}}(X)= {\mathrm {Set}}(Y) = {\mathrm {Set}}(Z)$ and *s*(*Y*) and *s*(*Z*) are the two children of *s*(*X*) in *S*.
2. *Dup*, then *v* is a binary node with two children corresponding to syntenies *Y* and *Z* such that ${\mathrm {Set}}(Y)= {\mathrm {Set}}(X)$, ${\mathrm {Set}}(Z) \subseteq {\mathrm {Set}}(X)$ and $s(X)=s(Y)=s(Z)$.
3. *pLoss*, then *v* is a unary node with a child corresponding to a synteny *Y* such that ${\mathrm {Set}}(Y) \subsetneq {\mathrm {Set}}(X)$ and $s(X) = s(Y)$.

If no ambiguity on the synteny of *v*, we will denote by ${\mathrm {Set}}(v)$ the range set of the synteny at node *v* of tree *T* (in other words, $\mathrm{Set}(v) = \mathrm{Set}(synteny(v))$.

An Unordered Super-Reconciliation (USR) $R_u({\mathcal {G}},S)$ of a set ${\mathcal {G}}= \{T_1, T_2, \cdots , T_t\}$ of gene trees with a species tree *S* is a labeled synteny tree which is an extension of the trees $\tilde{T}_i$, for $1 \le i \le t$, representing a valid unordered evolutionary history for ${{\mathcal {X}}}$. The cost $d(R_u({\mathcal {G}},S))$ of such an unordered Super-Reconciliation is the number of induced *Dup*, *fLoss* and *pLoss* events.

The unordered super-reconciliation problem then consists in inferring the USR of minimum cost. Notice that, as gene order is ignored, at most one *pLoss* can separate two binary nodes on a most parsimonious USR.

Regarding existence conditions, Lemma 2 and Theorem 1 clearly apply to the USR problem, as gene order information is not involved in tree consistency. Namely, an USR exists if and only if the trees of $\tilde{{\mathcal {G}}}$ are consistent, and in this case any supertree for $\tilde{{\mathcal {G}}}$ is the backbone of an USR. Lemma 3 and Lemma 4 also apply since, as mentioned before, their proofs do not involve gene orders. Therefore, we may assume that, if a supertree $\tilde{T}$ of syntenies is given, its nodes can be mapped according to the LCA-mapping and its speciation/duplication nodes identified in this way.

The USR problem thus reduces to a small phylogeny problem which consists in inferring internal node gene contents of the supertree $\tilde{T}$ leading to a minimal duplication and loss cost. As duplications are already determined by the node labeling of $\tilde{T}$, only loss events remain to be minimized. Notice that the root’s gene content is just ${\mathcal {F}}$.

We add to $\tilde{T}$ the *fLoss* branches obtained from the LCA-Reconciliation of $\tilde{T}$ with *S*. In other words, the new tree is an intermediate between $\tilde{T}$ and $R_u({\mathcal {G}},S)$. For practical reasons, we still call it $\tilde{T}$. Notice that *fLoss* branches can only create speciation nodes. For the requirements of the following algorithms, the empty synteny is assigned to the leaf created by an *fLoss* branch.

We now present a dynamic programming algorithm to find the minimum number of *pLoss* events required for a USR.

### A dynamic programming approach for optimal USRs

Given an USR *R*, we denote by $\mathrm{Set}_R(v)$ the range set of the synteny assigned to *v* in *R*. For an internal node *v* of $\tilde{T}$, denote

$$\begin{aligned} lca_{Set}(v) = \bigcup _{l \in {{\mathcal {L}}}(\tilde{T}[v])} \mathrm{Set}(l) \end{aligned}$$

as the set of all gene families that appear in a synteny under *v*. Note that *v* must have a gene in every family in $lca_{Set}(v)$. For a range set *X*, denote by $C_X(v)$ the minimum cost of an USR *R* between $\tilde{T}[v]$ and *S* in which we assign $\mathrm{Set}_R(v) = X$ (if $lca_{Set}(v)$ is not a subset of *X*, put $C_X(v) = \infty$). We denote $C_{lca}(v) := C_{lca_{Set}(v)}(v)$, i.e. the cost when we assign the smallest possible range set to *v*. If *v* is a leaf, we have $C_{lca}(v) = 0$ and $C_X(v) = \infty$ for any $X \ne Set(v)$. The value we are interested in is $C_{lca}(r)$, where *r* is the root of $\tilde{T}$.

We first show that the exact nature of the “extra” content that might be assigned to an internal node *v* is irrelevant for the computation of the optimal cost.

#### **Lemma 5**

  *Let*$v \in V(\tilde{T})$*be an internal node of*$\tilde{T}$, *and letX*, *Ybe any range sets satisfying*$lca_{Set}(v) \subsetneq X, Y$. *Then*$C_X(v) = C_Y(v)$.

#### *Proof*

This can be shown by induction on the depth of the nodes of $\tilde{T}$. The lemma is true for leaves, as $C_X(v) = C_Y(v) = \infty$ in this case. So assume that *v* is an internal node. Let *R* be a minimum USR of $\tilde{T}[v]$ in which $\mathrm{Set}_R(v) = X$. We build a reconciliation $R'$ in which $\mathrm{Set}_{R'}(v) = Y$. Consider the children $v_1$ and $v_2$ of *v* in $\tilde{T}$. If $\mathrm{Set}_R(v_1) = lca_{Set}(v_1)$, then either there is a *pLoss* node on the $vv_1$ path in *R*, or *v* is a duplication and *X* was partially duplicated. In any case for our USR $R'$, we can use this *pLoss* or duplication to lose $Y \setminus lca_{Set}(v_1)$, and assign $\mathrm{Set}_{R'}(v_1) = lca_{Set}(v_1)$ without incurring additional cost compared to *R*. We then use the same reconciliation as *R* for the subtree $\tilde{T}[v_1]$, and thus $R'$ incurs no extra cost on the $v_1$ side. If instead $\mathrm{Set}_R(v_1)$ strictly contains $lca_{Set}(v_1)$, then in $R'$ we put $\mathrm{Set}_{R'}(v_1) = Y$ without requiring any *pLoss* on the $vv_1$ path. Moreover by induction, $C_{\mathrm{Set}_R(v_1)}(v_1) = C_Y(v_1)$. It follows that $R'$ has as many losses as *R* in the $\tilde{T}[v_1]$ subtree — and since there is no loss on the $vv_1$ branch, $R'$ has at most as many losses as *R* on the $v_1$ side (note that $R'$ could have strictly less losses than *R* on the $v_1$ side if *R* had a loss on the $vv_1$ branch — this implicitly means that *R* had no such loss, as otherwise our $R'$ will end up having less losses than *R* and contradict its optimality). Now, it suffices to observe that the same scheme can be applied to $v_2$ as well (noting that losses saved by partial duplications cannot happen on both sides of *v*), showing that there is an $R'$ that is of cost no more than *R*. $\square$

Lemma 5 implies that there are two possible minimum loss costs for $\tilde{T}[v]$. Either *v* is assigned $lca_{Set}(v)$ and its cost is $C_{lca}(v)$, or it is assigned *X* with extra content and its cost is $C_X(v)$, for any *X* strictly containing $lca_{Set}(v)$. We will therefore denote by $C^*(v)$ the minimum loss cost of $\tilde{T}[v]$ when *v* is assigned any *X* such that $X \ne lca_{Set}(v)$. This leads to a dynamic programming formulation that takes into account the two cases. For an internal node *v*, define $spec(v) = 1$ if *v* is a speciation, and $spec(v) = 0$ otherwise. The value of $C^*(v)$ can be computed according to the following Lemma.

#### **Lemma 6**

*For an internal nodevof*$\tilde{T}$*with children*$v_1$*and*$v_2$, *we have*

$$\begin{aligned} C^*(v) = \min {\left\{ \begin{array}{ll} C_{lca}(v_1) + C_{lca}(v_2) + 1 + spec(v), \\ C^*(v_1) + C_{lca}(v_2) + spec(v), \\ C_{lca}(v_1) + C^*(v_2) + spec(v), \\ C^*(v_1) + C^*(v_2). \end{array}\right. } \end{aligned}$$

#### *Proof*

Let *R* be an optimal USR in which $\mathrm{Set}_R(v) = X \ne lca_{Set}(v)$. It follows that $X \ne lca_{Set}(v_1)$ and $X \ne lca_{Set}(v_2)$. The lemma lists all the possible ways of sending extra content to the children or not. In the first case, we have two losses: we lose $X \setminus lca_{Set}(v_1)$ and $X \setminus lca_{Set}(v_2)$ on both the $vv_1$ and $vv_2$ branches, respectively (if *v* is a duplication, we can save one loss in a partial duplication, hence the *spec*(*v*) term). In the second and third cases, we lose content only on one side (again potentially using a partial duplication), and in the last case, we transfer *X* to the children without incurring any loss. $\square$

The computation of $C_{lca}(v)$ has more cases, depending if the children of *v* have the same $lca_{Set}$ value or not. We show in Algorithm 1 how $C_{lca}(v)$ can be computed. We omit the proof of correctness for this algorithmn as it is similar to that of Lemma 6. 
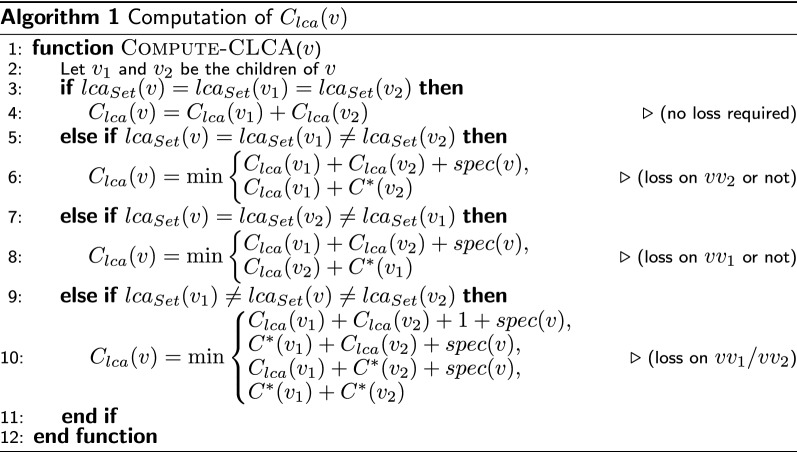


It is now clear that if the values of $C_{lca}(v_i)$ and $C^*(v_i)$ are known for the children $v_1, v_2$ of *v*, then $C_{lca}(v)$ and $C^*(v)$ can be computed in constant time, assuming we have access to $lca_{Set}(v)$ for every $v \in \tilde{T}$. By computing these values in a post-order traversal of $\tilde{T}$, we can compute $C_{lca}(r)$ for the root of $\tilde{T}$ in time $O(|V(\tilde{T})|)$. It is also straightforward to conceive a backtracking procedure to construct an actual USR. Moreover, every optimal solution can be produced by our dynamic programming paradigm.

This algorithm requires computing $lca_{Set}(v)$ for every vertex, which can be accomplished in time $O(|V(\tilde{T})||{\mathcal {F}}|)$ (recall that ${\mathcal {F}}$ is the set of gene families). This actually dominates the running time.

#### **Theorem 5**

*The minimum cost of a USR can be obtained in time*$O(|V(\tilde{T})||{\mathcal {F}}|)$.

## Application

### Simulated datasets

The dynamic programming algorithm that ignores rearrangements has been implemented in C++^1^ and tested on balanced trees obtained from simulated evolutionary histories. Simulations have been performed according to five parameters: *t*, the number of gene families in the ancestral synteny; *d*, the maximum depth of the balanced tree; $p_{dupl}$, the probability for any given node to be a segmental duplication; $p_{loss}$, the probability for a loss to occur under any given node; and $p_{length}$, the probability to remove one gene in a segmental loss, defining the probability for a loss to remove *k* genes (for $k \in \{1, 2, 3, ..\}$): $P(X = k) = (1 - p_{length})^{k - 1}p_{length}$, following a shifted geometric distribution.

Simulations yield Super-Reconciliations leading to fully labelled trees. The input of the Super-Reconciliation algorithm is then obtained from those trees by removing loss nodes and synteny information on the internal, non-root nodes.

From an accuracy point of view (results not shown), as expected the larger the density of duplication and loss events, the further is the simulated history from a most parsimonious history, and thus from the inferred tree.

**Fig. 2:**
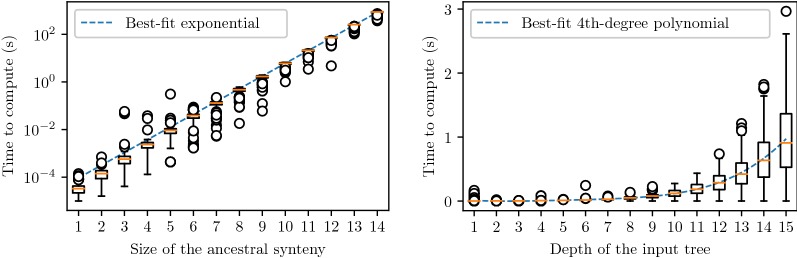
Time-efficiency of the algorithm with respect to the size of the ancestral synteny (for $d = 5$) and the depth of the input tree (for $t = 5$), for $p_{dupl} = p_{loss} = p_{length} = 0.5$. Note that the leftmost graph uses a logarithmic scale

As for time-efficiency, values for inferring the Super-Reconciliation of a single tree, aggregated over 500 simulations per value of *t*, the size of the ancestral synteny (number of gene families), are given in Fig. 2. Computations have been done on the “Cedar” cluster of Compute Canada with 32 *Intel 8160* CPUs operating at 2.10 GHz. As expected, running time exponentially increases with respect to parameter *t*. This prevented us from extending the simulations beyond an ancestral synteny of size 14, for which the Super-Reconciliation of a single tree of depth 5 required around 15 min. However, if the synteny size remains fixed, running times increase polynomially with the size of the trees. As shown by the right diagram of Fig. 2, for an ancestral synteny of size 5, simulations exhibit a running time of no more than few seconds for trees with depth up to 15, representing balanced trees with up to $2^{15}$ leaves.

Apart from genomic segments related through a recent whole genome duplicatiom event, real biological datasets are more likely to reveal large gene families rather than large sets of gene families evolving in concert. Thus, the increase in running time according to the size of the ancestral synteny is unlikely to be a bottleneck towards applying our Super-Reconciliation algorithm. The particular case of whole genome duplication is however worth exploring in more details.

### The opioid system

The opioid receptors, important regulators of neurotransmission and reward mechanisms in mammals, offer an interesting proof of concept, as these genes are present in clusters with conserved synteny in vertebrate genomes.

Three genes for the opioid receptors (OPR) were identified and named OPRD1 (delta), OPRK1 (kappa) and OPRM1 (mu). A fourth gene was later found (OPRL1) in rodents and human. In human, they are located on chromosomes 1, 6, 8 and 20.

**Fig. 3:**
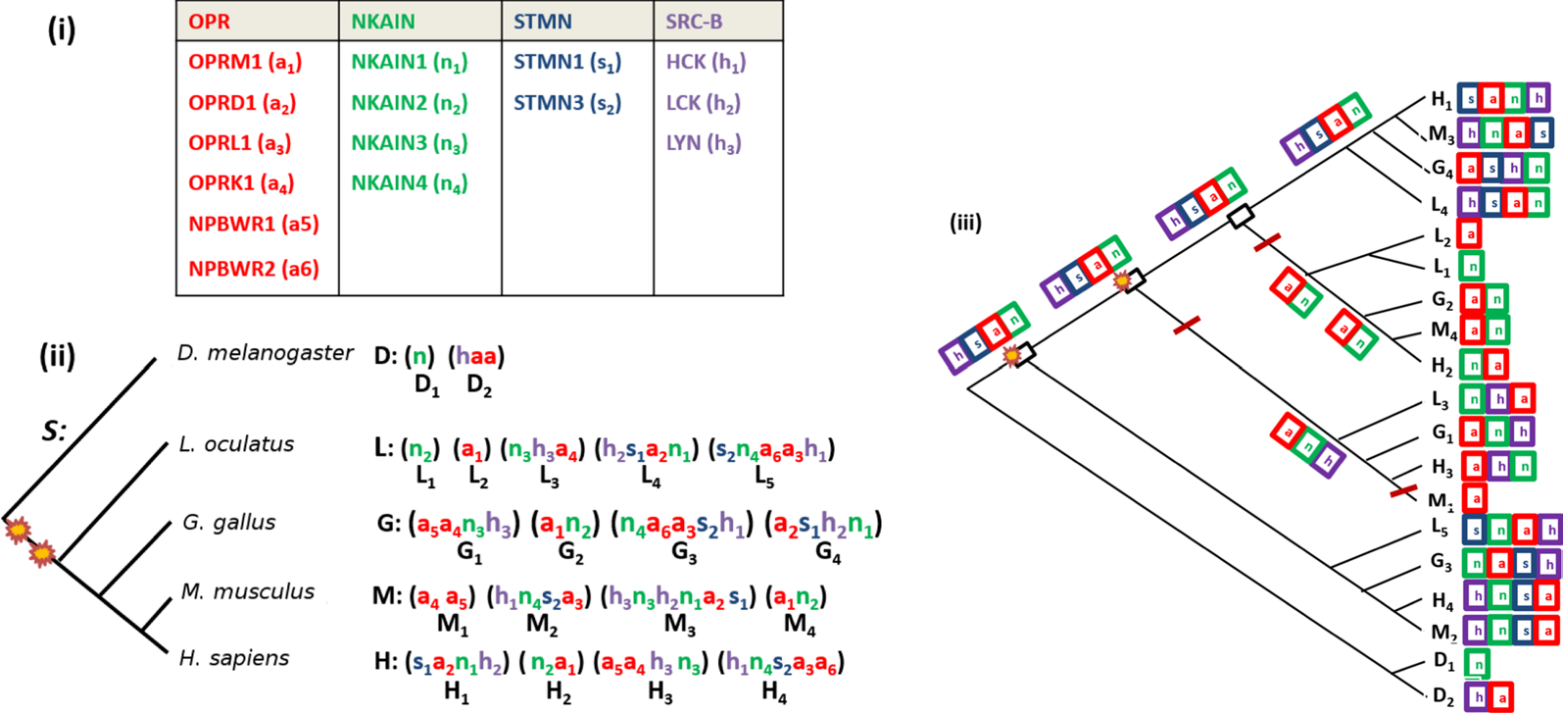
(i) The four considered gene families. (ii) The considered species tree with the corresponding clusters: 19 in total involving 24 genes from the OPR family (genes named ‘a’), 17 from the NKAIN family (named ‘n’), 7 from the STMN family (named ‘s’) and 13 from the SRC-B family (named ‘h’). (iii) The Super-Reconciliation obtained form individual gene trees (not shown), and the induced duplication and loss history. Losses are indicated by red bars on the considered edges and duplications by rectangles. Yellow stars indicate the location of the 1R and 2R whole genome duplication events. Gene orders after removing duplicates (see text) are indicated on leaves, and chosen gene orders for internal nodes are shown

Previous studies have considered the duplication scenario explaining the evolution of the opioid receptor genes [18–20]. The main question was whether observed paralogons arose from the two whole genome duplication events, often called 1R and 2R, known to have occurred early in vertebrate evolution.

By exploring regions surrounding the OPR genes in human, four syntenic regions, containing genes from three other families (NKAIN, SRC-B and STMN) apparently sharing a common history, were identified. From the analysis of individual gene trees (neighbor-joining and quartet-puzzling maximum likelihood trees), conclusions associating the evolution of the opioid system related genes to the 1R and 2R events were drawn.

Here, we consider the same four gene families OPR, NKAIN, STMN, and SRC-B, and further extend the OPR family with two neuropeptide NPBWR receptors, known to be closely related to the opioid receptors (Fig. 3(i)). Protein sequences and gene orders were downloaded from the Ensembl database (Release 92)^2^ for the following five species: *Homo sapiens*, *Mus musculus*, *Gallus gallus*, *Lepisosteus oculatus* (spotted gar) and *Drosophila melanogaster*. Gene orders are given in Fig. 3(ii).

For each gene family, we built a multiple sequence alignment with ClustalW [43] (Gonnet weight matrix and gap opening and extension penalties respectively set to 10 and 0.2). Maximum likelihood gene trees were subsequently constructed for each family using MEGA7 [44] (Jones-Taylor-Thornton substitution matrix and uniform rates among sites). As some syntenies contained paralogs (multiple copies from the same gene family, for example synteny $H_3$ contains two ‘*a*’), duplicates were removed so as to maximize gene tree consistency. Although gene trees were still inconsistent, the overall clustering of gene copies was preserved among gene trees, and consistency could be attained after some local adjustments, using the species tree as reference.

The obtained Super-Reconciliation is given in Fig. 3(iii). Notice however that gene orders are far from being consistent. In fact, all considered genomes are separated by a considerable evolutionary distance, and therefore, local rearrangements could have occurred along each lineage-specific branch. Choosing the (*h*, *s*, *a*, *n*) order on every node of the tree and assuming rearrangements to occur at terminal edges, i.e. after duplication and loss events, leads to a history of three duplications and two losses before the speciation of bony fish and tetrapods, with two duplications correlating with the 1R and 2R tetraploidization events. This result is in agreement with previous studies on the opioid receptor genes [18].

Further analysis, using more genes and species, is required to provide a more detailed scenario for the evolution of the opioid receptor genes. Our objective here however, was not to verify a given hypothesis, but rather to provide a proof of concept and explore the applicability and limitations of the proposed reconciliation model on real data.

## Conclusion

We have presented a natural extension of the DL Reconciliation model to handle segmental duplications and losses. This is the first effort towards developing a unifying automated method framework for reconciling a set of gene trees. We provide computational complexity results and a general inference method.

However, as the considered evolutionary model is restricted to losses and transposed duplications, the possibility of application to real datasets remains very limited. In particular, a duplication and loss history does not always exist for a set of syntenies if rearrangements are ignored, as the corresponding gene orders may be inconsistent. One solution would be to minimally correct gene orders to ensure consistency, before applying the DL Super-Reconciliation model. In this paper, we have considered an alternative way of working around this problem, which consists of extending the evolutionary model to account for rearrangements, but still only minimize duplication and loss events. The underlying Unordered Super-Reconciliation problem has been shown tractable. However, this way of integrating rearrangements is far from being fully satisfactory as the obtained evolutionary history may lead to a prohibitive number of rearrangements. In other words, the problem of Super-Reconciliation with rearrangements remains open.

Another strong constraint is the fact that tandem duplications, leading to syntenies with multiple gene copies, are ignored. In fact, only transposed duplications, i.e. duplications creating new syntenies, are allowed. Although the Super-Reconciliation model can easily be extended to tandem duplications by allowing for unary duplication nodes, the inference methodology developed in this paper is hardly applicable in this context. In particular, gene order consistency is a more challenging problem in presence of interleaving tandem duplications. In addition, having many gene paralogs in syntenies lead to multi-labeled trees (or mul-trees), i.e. trees with many leaves with the same label. This raises the issue of finding an appropriate definition of mul-tree consistency of a “mul-supertree”. But more importantly, what would be the meaning of a synteny mul-supertree, with the same synteny labeling more than one leaf? Clearly such a supertree cannot represent the backbone of a “valid” evolutionary history represented by a Super-Reconciliation.

A way of getting around this problem would be to prune multi-labeled gene trees in a way leading to “optimal” single-labeled trees. The more natural way to state the decision problem is whether there exists a way of choosing a single gene copy from each family represented in a synteny in such a way the obtained single-labeled gene trees are consistent. This is the way we implicitely handled the gene families of the opioid system. Alternatively, we can consider the optimization problem of finding the pruning minimizing a Robinson-Foulds distance between trees. Although authors have considered similar problems for mul-trees [45–48], as far as we know, none have yet handled these particular ones, representing an interesting avenue for future developments.

## Supplementary information

**Additional file 1.** The proof of NP-hardness of the Super-Reconciliation problem.

## Footnotes

1 The program and simulations are available at: https://github.com/UdeM-LBIT/SuperReconciliation

2 https://useast.ensembl.org/index.html.
