# Supplementary material for: Evolution through segmental duplications and losses: a Super-Reconciliation approach
Source: Algorithms Mol Biol. 2020 May 26;15:12. doi: 10.1186/s13015-020-00171-4 (PMC7249433; doi:10.1186/s13015-020-00171-4)
Supplement: Supplementary file 1 — Additional file 1. The proof of NP-hardness of the Super-Reconciliation problem. [file 13015_2020_171_MOESM1_ESM.pdf]

Additional file

NP-hardness of the Super-Reconciliation problem

We show here that finding a super-reconciliation that minimizes the number of Dup, $fLoss$ and $pLoss$ events is NP-hard. We reduce the problem of CUBIC 3-EDGE-COLORING to SUPER-RECONCILIATION. Given a graph $G = (V, E)$ in which each vertex has exactly 3 neighbors, the cubic 3-edge-coloring problem asks whether there exists a *proper coloring* of E with 3 colors, i.e. a partition of E into 3 sets $\{E_1, E_2, E_3\}$ such that for any vertex $v \in V$, the three edges incident to v all belong to a different E_i set. Note that if such a coloring exists, $|E_1| = |E_2| = |E_3| = |E|/3$. This problem was shown to be NP-hard in [49].

In what follows, for an integer k we will denote $[k] = \{1, 2, \ldots, k\}$. The father of a node v in a tree will be denoted $p(v)$ (p for 'parent'). Let $G = (V, E)$ be an instance of CUBIC 3-EDGE-COLORING, and denote $V = \{v_1, \ldots, v_n\}$. The ordering of the v_i vertices is not important but must remain fixed for the duration of the proof. To describe our corresponding SUPER-RECONCILIATION instance, we first define the species tree S, which is illustrated in Figure 4. Let S' be a caterpillar on leafset $V \cup \{\alpha, \beta, \gamma\}$ (here a caterpillar is a binary rooted tree in which each internal node has at least one child that is a leaf), where the leaves appear in the order $(\alpha, v_1, v_2, \ldots, v_n, \beta, \gamma)$ when traversing from the deepest to the closest leaf to the root. The species α, β and γ are special species, and the $v_1, \ldots, v_n$ species are those corresponding to V. For each $i \in [n]$, denote $p_i := p(v_i)$, and $p_0 := \alpha, p_{n+1} := p(\beta)$. To obtain S, for every $i \in [n+1]$, graft a large number of new leaves, say n^{10}, on the branch $p_{i-1}p_i$. Thus there are now n^{10} new internal nodes on the path between p_{i-1} to p_i, and we denote this set of n^{10} internal nodes as W_i, and the set of n^{10} newly inserted leaves as W_i^{leaf} (Figure 4 only shows 5 of the W_i and W_i^{leaf} nodes, with W_2 and W_2^{leaf} shown explicitly). Note that S is a caterpillar with $(n+1)n^{10} + n + 3$ leaves.

Now, denote $E' = \{(v_i, v_j), (v_j, v_i) : \{v_i, v_j\} \in E\}$, where we think of E' as the set of edges E, but where each edge appears in both directions. We define the set of syntenies (the content of these syntenies will be defined later — we are only listing them here)

$$\mathcal{X} = \{X_\gamma\} \cup \{X_{\alpha_i}, X_{\beta_i} : 1 \leq i \leq 3\} \cup \mathcal{X}_{E'} \cup \bigcup_{i \in [n+1]} \bigcup_{j \in [3]} \mathcal{X}_{i,j}^{leaf}$$

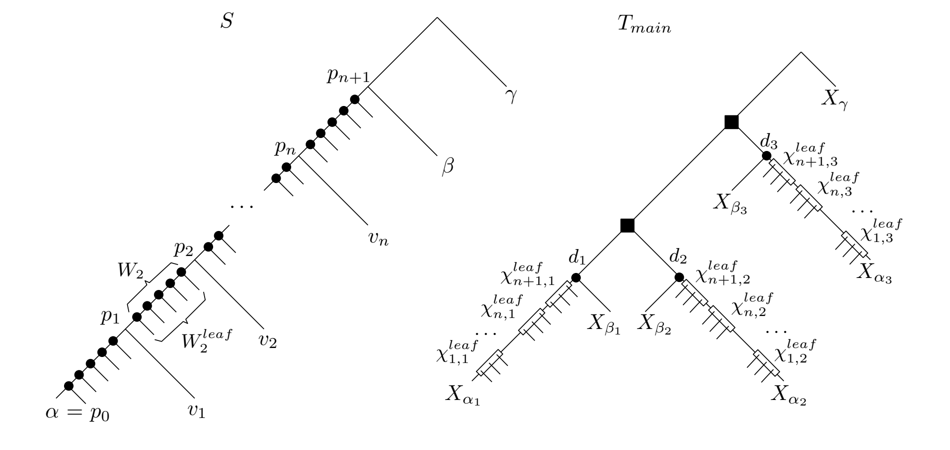

Figure 4 The species tree S constructed in our reduction and the main synteny tree T_{main}. The $\mathcal{X}_{i,j}^{leaf}$ syntenies of T_{main} refer to the leaves, not the internal nodes. The black squares indicate duplications.

where $\mathcal{X}_{E'} = \{X_{ij} : (v_i, v_j) \in E'\}$ and for each $i \in [n+1], j \in [3]$, $\mathcal{X}_{i,j}^{leaf}$ is a set of n^{10} syntenies that has exactly one synteny for each member of W_i^{leaf}. We put $s(X_{\alpha_i}) = \alpha, s(X_{\beta_i}) = \beta$ for each $1 \leq i \leq 3$, $s(X_\gamma) = \gamma$ and $s(X_{ij}) = v_i$ for each $(v_i, v_j) \in E'$. Hence the first subscript of a X_{ij} synteny indicates its species. Thus each species has 3 syntenies, except γ which has one. Observe that $|\mathcal{X}_{E'}| = 3n$.

Instead of describing the set of input gene trees, we describe them as synteny trees directly. We thus omit the usual tilde symbol, e.g., we write T instead of $\tilde{T}$ with the understanding that T is a synteny tree whose corresponding gene tree has a unique gene at each of its leaves. There is one main synteny tree T_{main}, illustrated on Figure 4. To obtain it, for each $j \in [3]$, define a tree T_{main}^j as the unique synteny tree on leafset $\{X_{\alpha_j}, X_{\beta_j}\} \cup \bigcup_{i \in [n+1]} \mathcal{X}_{i,j}^{leaf}$ that has only speciations. Another way to view T_{main}^j is that it is obtained by taking a copy of S, by removing the $v_1, \ldots, v_n$ species and relabeling the species at the leaves by the appropriate syntenies for the index j. The tree T_{main} is then obtained by joining the T_{main}^j trees and X_γ as follows (using Newick notation):

$$T_{main} = (((T_{main}^1, T_{main}^2), T_{main}^3), X_\gamma)$$

Note that T_{main} has 2 duplication nodes. We will call d_1 and d_2 the two children of the lower duplication, and d_3 the other duplication child (see Figure 4). The rough idea behind our reduction is that T_{main} has 3 subtrees that each contain a synteny from each species, except $v_1, \ldots, v_n$. These appear as losses in each T_{main}^j subtree. However, in $\mathcal{X}$ we have three syntenies for each species $v_1, \ldots, v_n$, just enough to "fill-up" these losses. The main goal in our construction is to make this complete "fill-up" of the losses possible if and only if G is 3-colorable by making each T_{main}^j subtree represents a color. We will build additional input trees to realize this idea. Our goal is to enforce that X_{ij} and X_{ji} can only fill-up the losses under the same subtree.

We have another synteny tree T'_{main} defined as:

$$T'_{main} = (((X_{\alpha_1}, X_{\alpha_2}), X_{\alpha_3}), X_\gamma)$$

Note that T'_{main} does not provide any new information on the structure of a supertree, but will play a role later in the gene ordering in the syntenies.

Then for each $v_i \in V$, let v_j, v_k, v_l be the neighbors of v_i and let

$$T_{ijk} = (((X_{ij}, X_{ji}), (X_{ik}, X_{ki})), X_\gamma)$$
$$T_{ijl} = (((X_{ij}, X_{ji}), (X_{il}, X_{li})), X_\gamma)$$
$$T_{ikl} = (((X_{ik}, X_{ki}), (X_{il}, X_{li})), X_\gamma)$$

For notational convenience, for all i, j, k we will say that $T_{ijk} = T_{ikj}$, i.e. both refer to the same tree. Note that for every edge $v_i v_j \in E$, X_{ij} occurs in exactly 4 trees (in 2 trees of the form T_{ijk} and 2 trees of the form T_{jik}). The set of synteny trees/families $\mathcal{G}$ contains T_{main}, T'_{main} and all the T_{ijk} trees.

It only remains to define the given gene orders for the extant syntenies $\mathcal{X}$. We will define these orders as strings on alphabet $\mathcal{G}$ directly. For $X \in \mathcal{X}$, let $t(X)$ be the set of synteny trees of $\mathcal{G}$ that X appears in. Observe that X_γ appears in every synteny tree, and thus there is only one possible ancestral sequence of gene families. In other words, the X_γ synteny is a string Z over alphabet $\mathcal{G}$, and the order of every other synteny X is the subsequence of Z for the characters $t(X)$. We let Z be any string that begins with T_{main} and ends with T'_{main}. This completes the construction of our Super-Reconciliation instance.

We show that G admits a proper 3-edge coloring if and only if our instance formed by $S, \mathcal{G}$ and order Z admits a Super-Reconciliation of total cost at most $3(n+1)n^{10} + 15n + 8$.

($\Rightarrow$) For the first direction, suppose that G admits a proper 3-edge coloring. Let E_1, E_2, E_3 be the underlying partition of the edges into 3 color classes. Let D_1, D_2 and D_3 be the subtrees of T_{main} rooted at d_1, d_2 and d_3, respectively. Notice that for each $l \in [3]$, the set $\{s(X) : X \in \mathcal{L}(D_l)\}$ is equal to $\mathcal{L}(S) \setminus V$. We "fill-up" each D_l subtree with a leaf from a synteny that belongs to v_j for every $v_j \in V$. More precisely, for each $l \in [3]$ and each $v_i \in V$, let $v_i v_j$ be the edge of E_l that is incident to v_i. We graft the corresponding synteny X_{ij} (from species v_i) onto D_l on the branch that makes its parent a speciation (this location is unique). Call T the synteny tree resulting from these graftings. In this manner, every D_l subtree has exactly one synteny from every v_i species. Moreover, every synteny gets grafted onto T, no new duplications are created and T has no full losses. It is not difficult to verify that the resulting tree displays all input trees (the T_{ijk} trees are displayed because two X_{ij} and X_{ji} syntenies will be grafted under the same D_l subtree, and this subtree is different from where X_{ik} and X_{ki} get inserted into, since $v_i v_j$ and $v_i v_k$ belong to a different E_l set).

Our tree T has 2 duplications and no full losses. To count partial losses, we assign the string Z to every internal node. Let X be any leaf of T that belongs to a species

in W_i^{leaf} for some $i \in \{1, \ldots, n+1\}$. The X synteny only appears in T_{main}, so because Z starts with T_{main}, we may add a single partial loss on the branch from X to its parent. This amounts to $3(n+1)n^{10}$ segmental losses. The syntenies X_{α_i} and X_{β_i} can be handled similarly with a single loss on the branch to their parent. As $i \in \{1, 2, 3\}$, this amounts to 6 losses. The X_γ leaf does not incur any losses. As for a synteny $X_{ij} \in \mathcal{X}_{E'}$, recall that X_{ij} appears in at most 4 gene families. This can be handled by using at most 5 segmental losses on the path between X_{ij} and its parent. As $|\mathcal{X}_{E'}| = 3n$, this adds at most $15n$ losses. In total, we the total cost is $3(n+1)n^{10} + 15n + 8$, which is conveniently the number that we predicted.

($\Leftarrow$) For the converse direction, let T be a supertree for $\mathcal{G}$ on leafset $\mathcal{X}$ that yields a reconciliation of cost at most $3(n+1)n^{10} + 15n + 8$. We assume that each internal node is assigned a gene family sequence that is a subsequence of Z.

Note that since T is compatible with T_{main}, T can be seen as a tree obtained by starting with T_{main}, then grafting some subtrees $T_1, \ldots T_r$ successively onto some branches of T_{main}. To see this, let T' be the tree obtained from T by deleting every node that does not have a descendant in $\mathcal{L}(T_{main})$. Then T' is the same tree as T_{main}, but with some nodes of degree 2 denoted $t_1, \ldots, t_r$ (excluding the root) that consist of the locations where the $T_1, \ldots, T_r$ trees were grafted (note that the roots of T_{main} and T must be the same due to X_γ). For each $i \in [r]$, we will assume that the root of T_i was grafted onto T_{main} under t_i, i.e. $r(T_i)$ is a child of the node t_i. Note that $\{\mathcal{L}(T_1), \ldots, \mathcal{L}(T_r)\}$ forms a partition of $\mathcal{X} \setminus \mathcal{L}(T_{main})$. Under the above view of T, we will think of a node x of T_{main} as also a node of T, whether x is internal or leaf.

We show how to obtain a proper edge coloring of G. The proof is divided into a series of claims, the first one showing that every leaf in a W_i^{leaf} must incur a segmental loss.

Claim 1 *Let $X \in \mathcal{X}_{i,j}^{leaf}$ be a synteny for which $s(X) \in W_i^{leaf}$ for some $i \in [n+1]$ and $j \in [3]$. Moreover let p_X be the parent of X in T_{main}. Then in T, there is either a duplication or a partial loss on the path from p_X to X.*

Proof Let Z_p be the gene family string assigned at p_X. Observe that in T, the p_X node has a descendant X_{α_i} for some $i \in [3]$. The family X_{α_i} appears in the trees T_{main} and T'_{main}, implying that both these families are in Z_p. Since X only appears in T_{main}, the T'_{main} character from Z_p must be lost on the path from p_X to X, either by a duplication or partial segmental loss. □

As a consequence of Claim 1, there are at least $3(n+1)n^{10}$ duplications and/or partial segmental losses in T. Our strategy is the following: we will show that if the X_{ij} syntenies are not setup to "fill up" every hole in the d_1, d_2 and d_3 subtrees of T as in our solution for the converse direction, then there must be at least n^{10} *full* losses in T (as opposed to partial losses). As these were not counted in Claim 1, this would imply that T has $3(n+1)n^{10} + n^{10} > 3(n+1)n^{10} + 15n + 8$ losses, a contradiction. We next show that the trees that get grafted onto T_{main} to obtain T all consist of syntenies from a single species.

Claim 2 *Let* $X_{ij} \in \mathcal{X}_{E'}$*, and let* T_h *be the tree grafted onto* T_{main} *that contains* X_{ij}*. If* T_h *has another leaf* $X_{kl} \in \mathcal{X}_{E'}$*, then* $k = i$*.*

Proof Suppose instead that $k \neq i$. Then $v_i = s(X_{ij}) \neq s(X_{kl}) = v_k$. We then have $s(r(T_h)) \geq lca_S(v_i, v_k)$. Assume without loss of generality that $k > i$. Observe that T_h cannot contain any leaf with a species in W_{i+1}^{leaf}, because all syntenies with a species in W_{i+1}^{leaf} are already in T_{main}. However, the path from $s(r(T_h))$ to v_i in S contains the set of nodes W_{i+1} (because $k > i$), where $|W_{i+1}| = n^{10}$. By the definition of reconciliation, in any reconciliation, each node in W_{i+1} must have at least one corresponding node in T_h on the path between $r(T_h)$ and X_{ij}, all of which must have a child that is a full loss in a node in W_{i+1}^{leaf}. It follows that T has at least n^{10} additional losses, a contradiction. □

Recall that T_{main} (and hence T) has two duplication nodes with children d_1, d_2 and d_3. Ignoring X_γ, these three nodes partition the leaves of T_{main} into 3 subsets. These will correspond to our edge colors. Towards this goal, for a synteny $X_{ij} \in \mathcal{X}_{E'}$, we will say that X_{ij} is of color 1 (respectively color 2 and 3) if it is a descendant of d_1 (respectively of d_2 and d_3). Note that X_{ij} has at most one color, but may have none - which we prove to not be the case.

Claim 3 *For each synteny* $X_{ij} \in \mathcal{X}_{E'}$*,* X_{ij} *has a (unique) color.*

Proof Suppose otherwise that there is some X_{ij} that has no color. Let T_h be the subtree grafted onto T_{main} that contains X_{ij}, with t_h the parent of $r(T_h)$. Since X_{ij} has no color, it follows that t_h must be an ancestor of d_1, d_2, d_3 or X_γ (all or some of these cases can hold simultaneously). In all cases, we have $s(t_h) \geq lca_S(\alpha, \beta)$ in any reconciliation. Moreover by Claim 2, T_h has only leaves from the v_i species. It follows that on the path from t_h to X_{ij}, there is a loss for each node in W_{i+1}. Once again, this incurs n^{10} additional losses, a contradiction. □

We then show that syntenies from the same species get distinct colors, owing to the T_{ijk} trees.

Claim 4 *Let* $X_{ij}, X_{ik} \in \mathcal{X}_{E'}$ *be two distinct syntenies from the same species* v_i*. Then* X_{ij} *and* X_{ik} *do not have the same color.*

Proof Suppose that X_{ij} and X_{ik} have color 1, without loss of generality. Let $T_{i'}, T_{j'}$ and $T_{k'}$ be the subtrees grafted onto T_{main} that contain X_{ij}, X_{ji} and X_{ik}, respectively (where $t_{i'}, t_{j'}, t_{k'}$ are the parents of $r(T_{i'}), r(T_{j'}), r(T_{k'})$, respectively). By Claim 2, we know that $T_{i'} \neq T_{j'} \neq T_{k'}$, although $T_{i'} = T_{k'}$ is possible. Recall that we have the tree $T_{ijk} = (((X_{ij}, X_{ji}), (X_{ik}, X_{ki})), X_\gamma)$ in the input. Since T displays T_{ijk}, this implies that $t_{k'}$ cannot be a descendant of $lca_T(t_{i'}, t_{j'})$, and therefore $T_{i'} \neq T_{k'}$. Also, because X_{ik} is of color 1, $t_{k'}$ must be a descendant of d_1. Thus $t_{k'}$ is either (1) an ancestor of $lca_T(t_{i'}, t_{j'})$, or (2) $t_{k'}$ is on the path between a leaf $w \in \mathcal{X}_{l,1}^{leaf}$ and its parent $p(w)$ in T_{main}, where $l \in \{1, \ldots, n+1\}$. In case (1), the only way that T can display T_{ijk} is if X_{ki} belongs to $T_{k'}$, along with X_{ik}.

This contradicts Claim 2. In case (2), let $T_{k''}$ be the subtree grafted on T_{main} that contains X_{ki}. Due to the T_{ijk} tree, $t_{k''}$ must also be on the path between w and $p(w)$. Thus in the subtree of T rooted at $lca_T(X_{ik}, X_{ki})$, there is at most one leaf other than X_{ik} and X_{ki} (namely w). This subtree must contain at least $n^{10} - 1$ losses, either for the W_{k+1} nodes if $i > k$, or the W_{i+1} nodes if $k > i$. We reach the same contradiction. □

It only remains to show that edge colors are consistent between their two directions.

Claim 5 *Let $X_{ij}, X_{ji} \in \mathcal{X}_{E'}$. Then X_{ij}, X_{ji} have the same color.*

Proof Let v_k, v_l be the neighbors of v_i other than v_j. Suppose that X_{ij} and X_{ji} do not have the same color. If one of X_{ij} or X_{ji} is of color 3 and the other of color 1 or 2, then because of the T_{ijk} tree in the input, X_{ik} and X_{ki} cannot be a descendant of any of d_1, d_2 or d_3. Hence they have no color, contradicting Claim 3. So we may assume that X_{ij} and X_{ji} are of color 1 and 2 (or vice-versa). Again because of the T_{ijk} tree, X_{ik} and X_{ki} must be of color 3. And because of the T_{ijl} tree, X_{il} and X_{li} must also be of color 3. But then, X_{ik} and X_{il} are both of color 3, contradicting Claim 4. □

We can now color the edges of E as follows: color v_iv_j with color $c \in \{1, 2, 3\}$ if and only if X_{ij} and X_{ji} have color c. By Claim 3 and Claim 5, each edge gets assigned a unique color. Two adjacent edges v_iv_j and v_iv_k get assigned the colors of X_{ij} and X_{ik}. By Claim 4, X_{ij} and X_{ik} have different colors. It follows that the edge coloring is proper, concluding the proof.
